# Design and Fabrication of a High Performance Microfluidic Chip for Blood Plasma Separation: Modelling and Prediction of System Behaviour via CFD Method

**DOI:** 10.1155/2023/3648247

**Published:** 2023-06-26

**Authors:** Hossein Amini, Amin Sokhansanj, Mohammad Akrami, Ismaeil Haririan

**Affiliations:** ^1^Chemical Engineering Faculty, Sahand University of Technology, P.O. Box 51335-1996, Sahand New Town, Tabriz, Iran; ^2^Reactor and Catalysis Research Center (RCRC), Sahand University of Technology, P.O. Box 51335-1996, Sahand New Town, Tabriz, Iran; ^3^Department of Pharmaceutical Biomaterials, and Medicinal Biomaterials Research Center, Faculty of Pharmacy, Tehran University of Medical Sciences, Tehran, Iran; ^4^Department of Pharmaceutics, Faculty of Pharmacy, Tehran University of Medical Sciences, Tehran, Iran

## Abstract

This paper presents a single-step microfluidic system designed for passive separation of human fresh blood plasma using direct capillary forces. Our microfluidic system is composed of a cylindrical well between upper and lower channel pairs produced by soft photolithography. The microchip was fabricated based on hydrophobicity differences upon suitable cylindrical surfaces using gravitational and capillary forces and lateral migration of plasma and red blood cells. The plasma radiation was applied to attach the polymeric segment (polydimethylsiloxane (PDMS)) to the glass. Meanwhile, Tween 80 was used as a surfactant to increase the hydrophobicity of the lateral channel surfaces. This led to the higher movement of whole blood, including plasma. Fick's law of diffusion was validated for this diffusion transfer, the Navier–Stokes equation was used for the momentum balance, and the Laplace equation was utilized for the dynamics of the mesh. A model with high accuracy using the COMSOL Multiphysics software was created to predict the capillary forces and chip model validation. RBCs (red blood cells) were measured by the H3 cell counter instrument, by which 99% plasma purity was achieved. Practically, 58.3% of the plasma was separated from the blood within 12 min. Correlation between plasma separation results obtained from software and experimental data showed a coefficient of determination equal to 0.9732. This simple, rapid, stable, and reliable microchip can be considered as a promising candidate for providing plasma in point-of-care diagnostics.

## 1. Introduction

Blood plasma is a primary source for the preparation of biological products. The presence of biomarkers in blood plasma has given its diagnostic value in the clinic. Plasma separation is a prerequisite for blood analysis in some diagnostic studies [1–3]. Differences in the particle sizes of blood and plasma have been used to separate blood components [4]. Several methods, such as centrifugation and hemapheresis, have been utilized to separate plasma from blood. However, conventional plasma separation processes have not been favorable for patients who require regular blood monitoring due to limitations of high cost, time-consumption, and inaccessibility [5]. The need for high blood volume and energy consumption is the other disadvantage of this method. So, simple and inexpensive alternative methods are needed for plasma separation. One of the new and efficient methods for separating blood compounds is using microfluidic devices with microfluidic-based systems, attempting to minimize the disadvantages of conventional methods [6–8].

It is well known that rapid blood tests at the beginning and over the course of treatment are significant. By using microfluidic device technology, not only has the whole blood analysis method been integrated into small devices but also the sample size, response time, and costs for large-scale production have been reduced [9]. Nowadays, two methods, named active [10] and passive [11] methods, have been reported for the development of chips to separate plasma, besides paper-based [12] and CD shape-based [13] microfluidic systems.

In an active strategy, the energy required for plasma separation is provided by the acoustic or electromagnetic fields, making the system more complicated. Active method-based devices have reduced the time needed for samples to reach the required situation. However, hydrodynamic forces and cell responses to various biophysical effects have been reported as the basis for passive-based devices. Nonstop operating, easy manufacturing, low cost, and simple design have been the factors that have led many researchers to focus on the importance of finding more efficient methods. Various techniques, including sedimentation, microfiltration, and hydrodynamic forces, have been used in passive devices based on the mechanical properties of particles. Passive devices have induced less stress on red blood cells than active ones, resulting in less hemolysis and more reliable diagnosis [14].

In addition, the necessity of a pump to inject blood samples into the device has been reported to be one of the major limitations of both active and passive devices. The major drawbacks reported in both studies were a low plasma recovery rate of about 3.4%, and challenging and expensive microchip construction. Some studies have used a vacuum desiccator method to inject the blood samples into the microchannel [15].

In the last decade, principles of capillary force, blood cell deposition, and cross-flow filtration have been applied to design and construct plasma separation by microfluidic systems. One study has extracted plasma by a “capillary flow and cross-flow filtration” method after PDMS modification of microfluidic channels by a surfactant without any external forces [16]. In another study, the asymmetric capillary flow of a microfluidic chip was designed through surface modification of the channel bonds by a multilayered spray coating of silica nanocrystals [17].

Some researchers have fabricated a bilayer PDMS microchip in which the upper membrane filter separates the plasma from the blood using gravimetric force [18]. In a similar study, a pump and vacuum were used to separate plasma and blood samples from the bottom of a PDMS cylindrical channel during plasma separation moved upwards. Meanwhile, the authors have used diluted blood as an inlet into the system. In addition, the time gap between the blood inlet in the first step and vacuum insertion in the final step showed the limited performance of the device. Valuable efforts have been made to develop a model for non-Newtonian fluid flow upon growing needs for diagnostic applications [19]. Liu et al. have interpreted the flow of non-Newtonian fluids through a direct channel using an altered Lucas–Washburn equation. Danilov et al. have investigated theoretical and experimental aspects of fluid flow through the capillary channels using non-Newtonian behaviour and a dynamic contact angle [20].

Some efforts have been made to commercialize microfluidic chips for plasma separation. CD-based microfluidic chips proposed centrifugal force for particle separation goals. Li et al. have designed the systems based on capillary and centrifugal forces and particle deposition, separating plasma with 99% purity [21].

Maria et al. designed a microfluidic chip with a wettability gradient and a cylindrical chamber that separated blood plasma after 15 minutes with a purification efficiency of about 99.9%. Using this system, variation of contact angle on the inner surface, self-built-in filter, and sedimentation resulted in plasma separation. They developed another microfluidic system-based plasma separation method to measure TSH (thyroid-stimulating hormone) levels using physical barriers proportional to blood and plasma particles. Finally, Liu et al. developed microchannels having both hydrophilic and hydrophobic properties to extract plasma with a purity of 85% in less than 10 minutes [22].

In addition, the importance of microfluidic chip modelling is due to the improvement of experimental performance constraints, optimization of the processes, and achievement of exact results. For example, Zhang et al. considered using a microfluidic chip with dielectric properties to classify blood cells based on the size to separate blood particles.

MATLAB and COMSOL software were used to calculate separation conditions, and in order to simulate the motion trajectory of cells in the microfluidic channel, the most effective parameters were selected [23].

Li et al. achieved 64% plasma separation by using a numerical technique to design a highly efficient microfluidic chip. Blood flow simulations were performed by a hybrid method of smoothed dissipative particle dynamics. The level of injected diluted blood and the speed of injecting blood in experiments have been declared by the immersed boundary method. The designed chip showed 40% efficiency in comparison with experimental plasma separation [24].

Shamloo et al. have presented a simple passive microfluidic device for blood plasma separation. Numerical studies and CFD simulation were used to solve the flow field, track the particles confined in it, and optimize channel dimensions and orientation angles. Utilizing optimization, they demonstrated that the performance of the device could be improved considerably, and an optimal design with a separation efficiency of 83% and a purity of 85% was achieved [25].

The purpose of the existing study was to extract the blood plasma for rapid diagnostic application at the point of care by designing a new surface-modified microfluidic chip through software simulation to predict and improve the performance of the device using the feedback from the experimental data. In this regard, for the recognition and prediction of the surface-modified microfluidic chip system from the CFD model, COMSOL metaphysics software was used. In addition, the CFD model designed based on fluid mechanics and mass transfer equations was validated by experimental data, and by using the validated model, the amount and purity of the extracted blood plasma were studied.

## 2. Research Methodology

### 2.1. Fabrication Procedure of Microchip

Standard soft lithography was utilized to fabricate the microfluidic devices as follows: a 180 *μ*m thickness was obtained by spin coating SU8-2050 (MicroChem, USA) at 1000 rpm for 30 seconds on a silicon wafer diced at 7 cm by 4 cm. It was patterned by our designed mask under UV light to create a master mold to construct the bottom channel. The polydimethylsiloxane (PDMS) base and curing agent were mixed at a ratio of 10 : 1 (w/w), which was followed by degassing in a vacuum jar for 30 min. It was then poured on the silicon mold using a Petri dish and heat-treated at 70°C for 3 hrs. After detaching the PDMS from the mold, both the inlet opening and the cylindrical wall were punched with a biopsy punch. The bottom surface of the PDMS was cleaned with adhesive tape and then treated with air plasma (2 min at 1 mbar) along with a glass slide for permanent bonding. Similarly, a master mold was prepared using the SU8-2050 (1700 rpm for 30 s) to construct the top channel with 100 *μ*m of thickness. After melding PDMS on the mold, the bottom surface of the top channel and the top surface of the bottom PDMS slab were exposed to air plasma. Then, they were bonded after aligning the top channel on the punched well. The whole device was finally cured at 100°C for 10 min. The change in different experiment parameters for the fabrication of microchips is shown in Table 1.

The cylindrical shaft part of the chip was fabricated of three sections: two hydrophilic upper and lower parts connected by one hydrophobic region in the middle. For this, the upper and lower segments were coated with Tween 80, while a small fraction of 1 mm at the top of the device (height) was covered by a rubber hose barrier (not coated). The plasma bonding was used for bonding the entire lower polymeric part to increase its hydrophobicity.

To measure the accuracy of blood volume, entering the inlet and also plasma volume, leaving the outlet, the whole blood was introduced into the device using a syringe pump at 0.8 *μ*L/min, so the entrance volume was measurable. A pipette tip was inserted in the outlet to collect plasma. By connecting the pipette tip to a measurable micropipette (0.1–10 *μ*L), it was possible to measure the plasma volume, easily by adjusting the gauge. Therefore, time, flow rate, and plasma volume were measurable using microsyringe device and micropipette accurately and precisely. In addition, the plasma purity was monitored by the H3 cell counter instrument.

**Figure 1:**
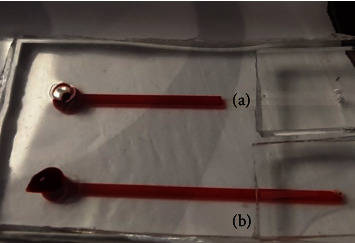
Photo of two experimental samples with (a) low and (b) high blood volumes which were not suitable for our plasma separation.

The device's inlet diameter as well as its height inlet was adjusted to control the initial driving force. While a low amount of blood entered via the inlet region, the blood flow stopped due to the coagulation phenomena (Figure 1(a)). Meanwhile, when high blood quantity entered via the channel inlet region (Figure 1(b)), it passed through the cylindrical shaft with no separation. After several attempts, the optimized quantity of 10 *μ*l blood was chosen as the appropriate amount of blood to be applied (3–5 droplets).

### 2.2. Tuning the Vertical Channel Height and the Duration of Bonding

The duration of plasma bonding was a very important factor when PDMS stuck to the silicon. The proper time for the operation was estimated to be 2 minutes. Furthermore, because the height of the cylindrical shaft depended on the height of the lower polymer block of the chip, the most appropriate size was determined to be between 4 and 5 mm, while exposure time was 2 min.

According to Table 2, by increasing the time of plasma bonding, the amount of hydrophobicity of the device was raised more than the desired limit for plasma separation (2 min), while the hydrophobic area in the middle of the cylindrical shaft was consumed at 1 mm. In optimal conditions (i.e., 2 min duration for plasma bonding and 4 mm of block height), a short distance was needed for blood to self-filtrate in the cylindrical shaft for the separation process.

**Figure 2:**
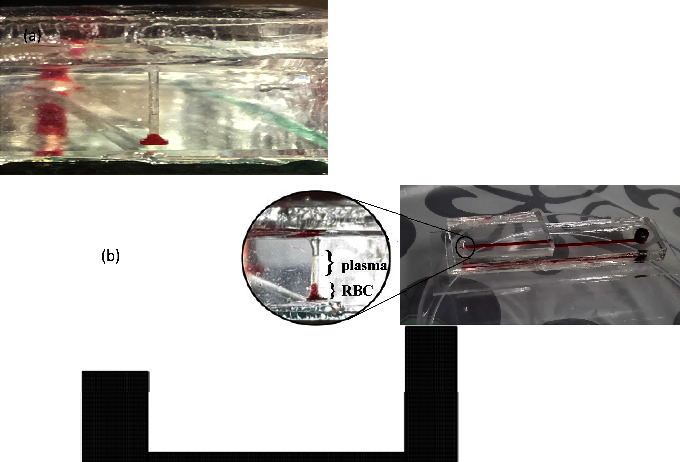
(a) Plasma separation section (cylindrical well) and (b) schematic representation of the microfluidic chip.

On the other hand, when the height of the polymer block was lower than the optimum size (4 mm), the plasma separation process became much more difficult due to its greater hydrophobicity. Conversely, when the polymer block height was more than the optimal size, the length of the hydrophilic increased, causing the whole blood to be removed. The separation section is shown in Figure 2(a).

### 2.3. Preparation of Blood Sample

The rheological properties of blood are affected by various environmental factors, such as temperature, pressure, and storage; blood should be freshly prepared before injection. Here, 10 *μ*l of blood were poured into the inlet opening channel using a syringe pump for the separation process. Before introducing the blood into the chip, the number of cells of each blood sample was counted using a cell counter for future comparison. To facilitate the quantification of cells by the cell counter, adding the minimum amount of anticoagulant to the samples was necessary. The results of four blood determinations (WBC, RBC, HGB, and HCT) from cell counter data were compared to separate plasma exiting from the outlet opening chip.

The amount of blood needed to be optimized, i.e., the plasma separation process is actually affected by both capillary force and surface tensions induced by fabricated variables. If not, the greater volume of blood will show initial force movement, which can prevent plasma separation in the cylindrical shaft.

### 2.4. Plasma Separation Mechanism and Measurements

The basis of separation in the cylindrical well part based on the schematic below (which includes two hydrophilic parts and one hydrophobic part at the well part and the horizontal hydrophilic part of the microchip) is that when the blood sample reaches the first hydrophilic part of the cylindrical well part, due to the capillary force of the surface of the microchip and the hydrophilic nature of the plasma, the microchip separates the plasma from the other particles of the blood components, and this force passes the plasma through the middle nonhydrophilic region and directs it to the second hydrophilic part and the outlet of the chip. Also, the particles of blood that remain at the beginning of the hydrophobic part act like a filter for other blood particles, and thus separation is done. In this work, to improve separation, in addition to making two parts of the well hydrophilic, we also made the horizontal part of the microchip hydrophilic using Tween 80, which helped the performance of the chip in separation. Before measuring the outlet fluid flow from the chip, two parameters were evaluated: (a) plasma appearance and (b) plasma purity. Plasma purity was determined by the cell counter using the following:

(1) $$\begin{array}{c} Plasma\;Purity=1-\left(\frac{number\;of\;blood\;cells\;in\;the\;output}{number\;of\;blood\;cells\;in\;input}\right)\text{.} \end{array}$$

Subsequently, to determine the volume of separated plasma from blood samples using a microfluidic chip at certain times, the COMSOL Multiphysics software was used.

### 2.5. Modelling

**Figure 3:**
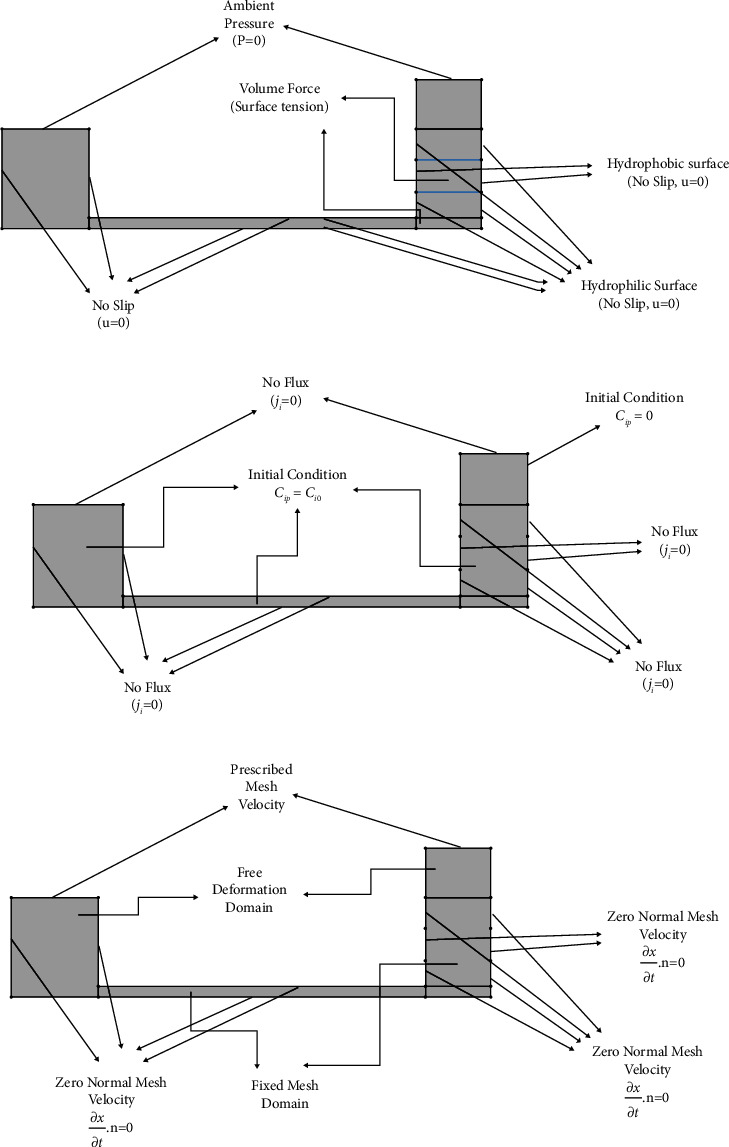
(a) Boundary and volumetric conditions of fluid flow motion, (b) boundary and initial conditions of plasma mass transfer, and (c) dynamic mesh boundary conditions.

The 2D geometry arrangement of the plasma separator microchip as shown in Figure 3 was modelled in the form of the continuous flow at the microchannel in COMSOL Multiphysics (v.5.5). The main benefit of the suggested model is to display how the fluid flows into the microchannel and predict the amount of plasma separation and plasma concentration profile at each moment of the process. By using the suggested model, the behaviour of the system can be studied with great accuracy. Finally, using equations (2) and (3), the dependent and average of the squares of the errors between the modelling and experimental data were determined.

(2) $$\begin{array}{c} R^{2}=\frac{\overset{n}{\underset{i=1}{\sum }}{\left(x_{i,\exp}-x_{avg}\right)}^{2}-\sum_{i=1}^{n}{\left(x_{i,\exp}-x_{i,Model}\right)}^{2}}{\overset{n}{\underset{i=1}{\sum }}{\left(x_{i,\exp}-x_{avg}\right)}^{2}}\text{,} \end{array}$$

(3) $$\begin{array}{c} MSE=\frac{\sum_{i=1}^{n}{\left(x_{i,\exp}-x_{i,Model}\right)}^{2}}{N}\text{.} \end{array}$$

#### 2.5.1. Governing Equations

The fluid flow in the microchannel was created under the influence of the capillary force of the microchannel hydrophilic part. The equations used for the modelling of the separation of blood plasma in the designed microchip are the continuity equation for the mass balance, the Navier–Stokes equations for the momentum balance, Fick's law equation for the mass transfer of the plasma, and finally, the Laplace equation for the dynamics of the mesh of the domain. The equations mentioned are listed in Table 3. In this table, *µ*_*l*_, *ρ*_*l*_, and $\overset{⟶}{v}$ represent the sample's viscosity, density, and velocity vector inside the microchip, respectively. Also, *D*_*i*_^*T*^, *D*_*i*_^*f*^, *ω*_*i*_, *M*_*i*_, *M*_*n*_, and *x*, respectively, represent the thermal diffusion coefficient of the sample components, the mass diffusion coefficient of the sample components, the mass fraction of the sample components, the molecular weight of the sample components, the average molecular weight of the sample, and the separated plasma displacement vector. The following assumptions were also considered to solve these equations in the generated geometry:

- The viscosity and density of the blood sample were considered constant in the entire microchannel (at the ambient temperature and pressure)
- The surface tension and contact angle of the blood sample plasma were considered constant in the hydrophilic area of the microchannel (at the ambient temperature and pressure)
- The fluid flow inside the microchannel is considered stationary
- The operating temperature of the solved model is assumed to be constant throughout the microchannel
- The diffusion coefficient of Fick's law equation for major components of the blood sample throughout the microchannel was supposed to be constant

#### 2.5.2. Boundary Conditions

All the boundary, initial, and volumetric conditions used to solve the partial differential equations mentioned in Figures 3(a)–3(c) are specified in full detail.

## 3. Results and Discussion

In this section, the results of microchip output as plasma separation efficiency were compared with the theoretical results obtained from modelling using the CFD method with COMSOL software. A batch system with a specific initial input value was used and designed to achieve plasma separation and microchip modelling.

### 3.1. Experimental Results

According to Table 1, method number 2 was selected because of its better efficiency in blood plasma separation. Therefore, this term formed the basis of simulation and manufacture.

#### 3.1.1. Plasma Separation Efficiency

The amount and purity of the separated plasma were considered as criteria for separation efficiency. For diagnostic applications, the purity of outlet plasma is very important, whereas for plasma production purposes, the amount of plasma is the critical point.

#### 3.1.2. Amount of Separated Plasma

In practice, after 12 minutes, the separated plasma was collected at the chip output by a syringe, and its volume was measured. However, to obtain the exact amount of separation based on time, COMSOL software was used. The output plasma volume at different times is reported in Table 4. At 12 minutes, the highest separation rate was recorded, which was 58.3% of the available plasma, equal to 3.5 microliters.

#### 3.1.3. Purity of Separated Plasma

The results obtained from the cell counter device before and after separation are reported in Table 5. Values of four parameters, including RBC, WBC, HGB, and HCT, were compared. As recorded, the RBC count (before separation) was 4.42 million and that of white blood cells was 7,000 per 1 *μ*l.

Considering the analysed data for inlet and outlet of blood particles obtained by H3 cell counter measurement, as shown in Table 5, plasma purity calculated by (1) was obtained as 99%. This high purity demonstrates the excellent microchip performance of plasma separation in this study.

### 3.2. Modelling Results

#### 3.2.1. Model Accuracy

**Figure 4:**
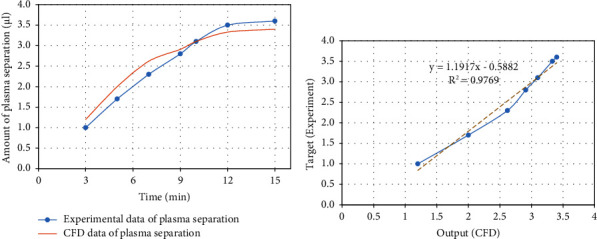
(a) Amount of plasma separation (ul) vs. time in experimental and modelling data and (b) correlation diagram between modelling and experimental data.

The results of Figure 4(b) showed the value of mean squared error (MSE) and coefficient of determination (*R*^2^) between the model and experimentally measured data. The mean squared error and the coefficient of determination were about 0.04585 and 0.9732, respectively, representing proper compatibility between the model and experimentally measured data. Therefore, the mathematical model of CFD can be utilized to predict the behaviour of the designed laboratory system, parametric study, and how blood plasma is separated.

To verify the CFD modelling with experimentally measured data, region size and boundary conditions were considered to be equal to the utilized one in the laboratory system. For comparison, the modelling data were compared to the laboratory data. As shown in Figure 4(a), the modelling outcomes were in high-grade accordance with the measurements gained experimentally, as demonstrated in Figure 4(b). Also, the difference between the values of experimental data and modelling results can be attributed to the error of measuring the data by the operator in the laboratory, the error due to the rapid coagulation of the blood sample in contact with the ambient air of the laboratory, and the error of numerical modelling, and simplifying assumptions.

#### 3.2.2. Evaluation of Velocity Profile of Sample and Plasma Concentration and Displacement Rate of Separated Plasma Inside the Microchip

**Figure 5:**
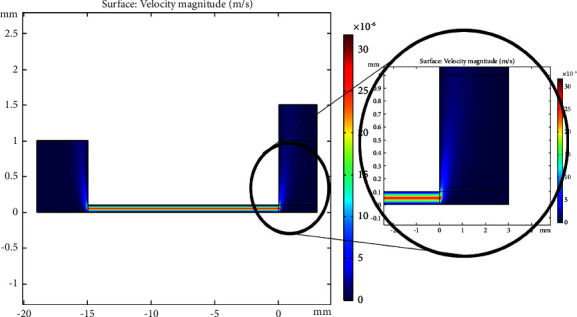
Velocity profiles of the sample inside the microchip.

**Figure 6:**
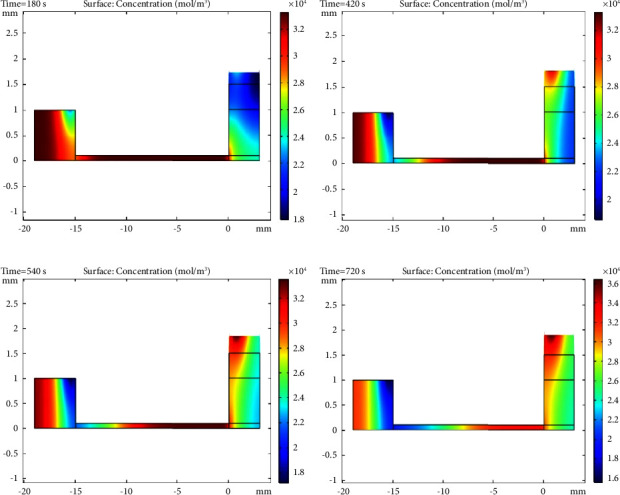
Plasma concentration profile of the sample inside the microchip (mol/m3) vs. time: (a) 3 min, (b) 7 min, (c) 9 min, and (d) 12 min.

**Figure 7:**
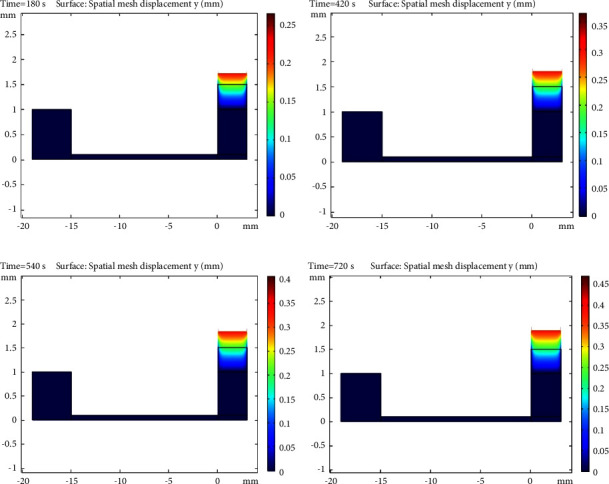
Displacement plasma of the sample inside the microchip (mm) vs. time: (a) 3 min, (b) 7 min, (c) 9 min, and (d) 12 min.

As mentioned in the previous sections, due to the excellent fitness between the laboratory and the modelling data, the steady-state velocity profile of the blood sample was obtained at different intervals of 3, 7, 9, and 12 minutes in Figures 5–7, respectively. As shown in Figure 5, the capillary force generated by the designed microchip causes suction of the plasma in the blood sample. This force creates convection mass transfer of the plasma and finally leads to movement of the plasma across the microchip towards the hydrophilic region and plasma separation. According to Figure 6, plasma concentrations throughout the microchip decreased over time due to convection and diffusion mass transfer into the hydrophilic region, and plasma concentrations in the hydrophilic region continuously increased. Finally, Figure 7 shows the displacement rate of separated plasma over time.

As seen, the height of the right side of the microchip increased over time, indicating the separation of plasma from the blood sample by the designed microchip. It should also be noted that the change in sample height at the entrance of the microchip was assumed to be negligible due to the large inlet diameter.

#### 3.2.3. Prediction of the Amount of Plasma Separated Using CFD Modelling

**Figure 8:**
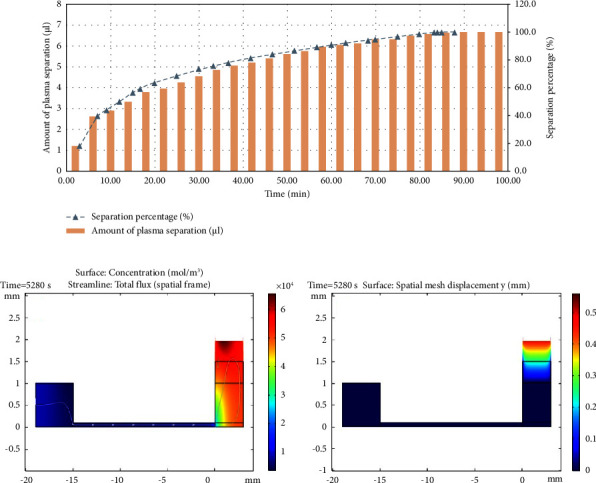
(a) Plasma separation percentage modelling data% vs. time (min), (b) plasma concentration profile of the sample inside the microchip (mol/m^3^) at the final time (88 min), (c) displacement of plasma of the sample inside the microchip (mm) at the final time (88 min).

As the CFD model was fitted to experimental data efficiently, the model was used to predict the end time of the complete plasma separation.  Figure 8 shows the percentage of separated plasma volume over time based on the CFD model. It is clear that in about 88 minutes, a relatively complete separation percentage (99.9%) can be achieved by the designed microchip. However, this long time is due to the coagulation that occurs along the path and reduces the flow rate. However, the highest separation rate was recorded at 12 minutes, which is enough for the subsequent analysis.

Taken together, in the study, a software simulation was used to predict and improve the performance of the microdevice for plasma separation based on the feedback from the experimental data. In addition to acceptable correlation between experimental and software data, better separation efficiency and separation time were concluded in comparison with published data, introducing a simple, rapid, and high-throughput device for diagnostic application in resource-limited environments and point-of-care settings such as measuring biochemical components and blood biomarkers can be measured.

## 4. Conclusions

In this design, we fabricated a novel microfluidic system and modelled this system using transport phenomena in COMSOL Multiphysics software for plasma separation. Simulated aspects and feedback were applied to improve device fabrication in terms of flow rate, hydrophobicity, channel length, input load, and reaching a separation efficiency of 99%. An acceptable correlation was obtained between experimental data and data obtained from the software. The separation time of 20 minutes reported by Maria et al. [22] was improved to 12 min in our microchip. Geometrically, the size of the lower part of the channel was decreased to get the desired separation condition through a cylindrical well. In comparison, the improved hydrophobicity of the horizontal channel increased blood flow to the cylindrical well, leading to decreased time of separation. Performing multiple tests to determine the system's performance is very time-consuming, costly, and has problems with regards to the preparation of blood samples and equipment. Hence, the process of separating plasma from blood samples was modelled using the CFD method based on transfer phenomena. The coefficient of determination (*R*^2^ = 0.9732) of the CFD model with Fick's law, Navier–Stokes equations, and the Laplace equation showed the suitability of the model to estimate and predict the value of separated plasma. Consequently, the suggested model can be applied to other complex microchips in different operating conditions. The development of such novel microchips can be helpful for plasma production and postchip plasma analysis in clinics. This simple, fast, stable, high-throughput, and reliable microchip can benefit for providing plasma in point-of-care diagnostics.

## Figures and Tables

**Table 1:** The change of different experiment parameters for the microchip fabrication method.

No.	Subsurface					Top surface					Mold	
	SU8 (rpm)	Basic baking (min)	Expose (s)	Later baking (min)	Hard baking (min)	SU8 (rpm)	Basic baking (min)	Expose (s)	Later baking (min)	Hard baking (min)	PDMS weight (gr)	Curing (cc)
1	1000	30	10	12	3	1600	20	10	10	5	21	0.097
2	1500	40	10	12	4	3000	20	10	10	4	21	0.18
3	1600	45	10	12	4	3000	25	10	11	4	21	0.17
4	1500	45	10	12	5	3000	20	10	12	4	21	0.2

Method number 2 was selected because of its better efficiency in blood plasma separation.

**Table 2:** Different states of the plasma bonding device.

Raw	Polymer block height (mm)	Plasma bonding time (min)	Performance
1	3	4	Unfavorable
2	5	4	Unfavorable
**3**	**4**	**2**	**Favorable**
4	6	2	Nearly unfavorable
5	4	5	Unfavorable
**6**	**4.5**	**2**	**Favorable**
7	4.5	4	Unfavorable
8	6	4	Unfavorable

States 3 and 6 were selected because of better performance.

**Table 3:** Governing and supplementary equations used in modelling.

Continuity equation	$\nabla .\left(\rho_{l}\overset{⟶}{v}\right)=0$
Momentum equation	$\nabla .\left(\rho_{l}\overset{⟶}{v}\overset{⟶}{v}\right)=-\nabla p+\nabla .\left(Ԏ\right)+\rho_{l}\overset{⟶}{g}$
	$Ԏ={µ}_{l}\left[\left(\nabla \overset{⟶}{v}+\nabla {\overset{⟶}{v}}^{T}\right)\right]$

Mass transfer equation	*∂*(*ρ*_*l*_*ω*_*i*_)/*∂*t+∇∙*j*_*i*_+*ρ*(*v*∙∇)*ω*_*i*_=0*j*_*i*_=−(*ρ*_*l*_*D*_*i*_^*f*^∇*ω*_*i*_+*ρω*_*i*_*D*_*i*_^*f*^∇*M*_*n*_/*M*_*n*_ − *j*_*c*.*i*_+*D*_*i*_^*T*^∇*T*/*T*)
	$M_{n}={\left(\underset{i}{\sum }\omega_{i}/M_{i}\right)}^{-1}$ $\begin{array}{c} j_{c.i}=\rho_{l}\omega_{i}\underset{K}{\sum }M_{i}/M_{n}D_{K}^{f}\nabla \omega_{K}\\ \nabla T=0 \end{array}$

Dynamic mesh equation	(*∂*^2^/*∂X*^2^)(*∂x*/*∂t*)+(*∂*^2^/*∂Y*^2^)(*∂x*/*∂t*)=0

**Table 4:** Plasma separation efficiency for different time.

Time (min)	Outlet plasma amount (*μ*l)
3	1
7	2.3
9	2.8
12	3.5

**Table 5:** Comparison of results between inlet fresh blood and outlet separated plasma by H3 cell counter.

Blood cells	Fresh blood	Separated plasma
WBC	7.11 *∗* 10^3^/*μ*l	7 *∗* 10^1^/*μ*l
RBC	4.42 *∗* 10^6^/*μ*l	4.34 *∗* 10^4^/*μ*l
HGB	12.2	0.006
HCT	39.9%	0.08%

## Data Availability

All the experimental data are included in the manuscript.
